# A Mixed Methods Design to Detect Adolescent and Young Adults' Impulsiveness on Decision-Making and Motor Performance

**DOI:** 10.3389/fpsyg.2019.01072

**Published:** 2019-05-24

**Authors:** Queralt Prat, Juan Andueza, Berta Echávarri, Oleguer Camerino, Tiago Fernandes, Marta Castañer

**Affiliations:** ^1^INEFC (National Institute of Physical Education of Catalonia), University of Lleida, Lleida, Spain; ^2^Universidad Pública de Navarra, Pamplona, Spain; ^3^Universitat de Lleida, Lleida, Spain; ^4^Faculty of Sport, Center for Research, Training, Innovation and Intervention in Sport, University of Porto, Porto, Portugal; ^5^IRBLLEIDA (Lleida Institute for Biomedical Research Dr. Pifarré Foundation), University of Lleida, Lleida, Spain

**Keywords:** impulsive actions, motor skills, behavior coding tool, T-pattern detection, methods integration

## Abstract

Impulsiveness in adolescents and young adults is an important aspect of psychological research. However, there still is lack of research that relates impulsiveness and motor performance in those. Thus, we aim to detect the levels of impulsiveness related to motor skills, motor laterality, spatial orientation, and individual interaction on the decision-making of adolescents and young adults across three staggered workouts. The study had 71 participants (53 males and 18 females), ranging in age from 17 to 24 years old (*M*age = 18.5 years; *SD* = 1.72) and classified as non-impulsiveness (*n* = 47), impulsiveness (*n* = 17), and attention deficit hyperactivity disorder (ADHD) (*n* = 7). A Mixed Methods research was conducted throughout four research tools (a) The Observational System of Motor Skills (OSMOS) was used to observe and detect the movement sequences patterns; (b) The Spanish version of Impulsive Behavior Scale (UPPS-P) was administered to obtain the impulsiveness profiles; (c) The Precision and Agility Tapping over Hoops (PATHoops) was carried out to observe the decision-making and temporal-spatial over movement effectiveness; (d) Motor Laterality Inventory (MOTORLAT) was applied to obtain the laterality profiles related to motor skills performance. This Mixed Methods approach has obtained useful results for impulsiveness in motor situations as the results from the different tools converge to established three clear profiles of impulsiveness. Participants with ADHD showed lack of interpersonal interaction, non-resolute decision-making, and lesser richness of motor skills patterns than non-impulsiveness and impulsiveness subjects. Additionally, impulsiveness participants also showed rich motor patterns, dyadic interactions, good decision making in spatial orientation tasks, and more versatile laterality in the lower limbs.

## Introduction

Impulsiveness is widely investigated in adolescents and young adults mostly related to different addictions such as gambling, tobacco, drugs, and alcohol consumption (e.g., Wiers et al., 2010; Franco et al., 2016; Secades-Villa et al., 2016). Also, researchers have studied the relationships between impulsiveness and aggressive characteristics, pursuing new sensations, and risk situations (Svebak and Kerr, 1989).

Adolescence is an essential stage in the development of a human being, characterized by the evolutionary moment that individuals should consolidate personality, and are most influenced by all kinds of stimuli. Delimited between by the ages of 12 and 25, adolescence is divided into three stages: (a) early adolescence: 12–14 years; (b) middle adolescence: 15–17 years; (c) late adolescence: 18–25 years (Spear, 2013). Therefore, young adults in their twenties are nowadays considered to be in late adolescence. During the adolescent period, the control of the impulses is still regarded as immature (Casey and Jones, 2010), meaning that not only teenagers but young adults look for new, risky, and impulsive sensations (Cyders and Smith, 2008). These are critical periods in ontogenesis, which the setting of habits unbalances the personal and social dimensions (Cerkez et al., 2015).

During the teen stages, impulsiveness is an issue commonly studied with substance addiction or abuse, attention problems and attention deficit hyperactivity disorder (ADHD). (Moeller et al., 2001:1784) define impulsiveness “*as a predisposition toward rapid, unplanned reactions to internal or external stimuli without regard to the negative consequences of these reactions to the impulsive individual or to others.”* The same authors recognize impulsiveness, not as an isolated act, but as a pattern of mechanisms that follow the succeeding phases: (a) *Predisposition* to react unexpectedly and quickly; (b) a fast *Response* without planning; (c) *Action* without considering the consequences.

Complementarily, the personality traits studied based on impulsiveness focus on (a) *Motor impulsiveness****:***thoughtless behaviors resulted from an immediate response to a stimulus; (b) *Attentional impulsiveness****:***low control over the intrusion of thoughts and difficulties for sustained care; (c) *Impulsiveness by unpredictability****:***precipitate processing of information that leads to quick and unplanned decisions (Patton et al., 1995; Barratt et al., 1997); (d) *The Quest for Adventure* (Eysenck and Eysenck, 1977) individuals are carried away by the moment without being aware of the risk of their actions; (e) *The Search of novelty* (Cloninger et al., 1993): personality trait associated to an exploratory activity as response to a new stimulation and usually focused on the rewards; (f) *The search for sensations* (Zuckerman, 1990, 2007):the need to experience diverse situations that arise the sense of physical and social risk. Although the impulsiveness in risky situations is associated with the scope of Physical Activity and Sport, the binominal between impulsiveness and motor performances is scarce (Svebak and Kerr, 1989).

### Impulsiveness, Decision-Making, and Goal Achievement

Dickman (1990, 2000) distinguishes two types of impulsiveness: dysfunctional impulsiveness and the functional impulsiveness. On the one hand, dysfunctional impulsiveness is disordered and unproductive behavior that does not have benefits for the teenagers and young adults. On the other hand, functional impulsiveness refers to the trend of acting rapidly with the possibility of committing errors in processing information, probably because of the inability to identify relevant elements for decision-making (Baker and Côté, 2003).

Moreover, functional impulsiveness is related to enthusiasm, risk-taking, and development of high levels of activity and audacity (Dickman, 1990, 2000). Aspects that, as a whole, represent a personality trait that allows the youngsters to process information quickly and effectively. Relating motor impulsiveness (Patton et al., 1995; Barratt et al., 1997) with functional impulsiveness (Dickman, 1990, 2000), in a Physical Activity and Sport point of view, emerges the relationship between rapid decision-making capability in the face of a stimulus (Burnett et al., 2012) and the ability to motor anticipation (Murgia et al., 2014). We consider that this relationship is fundamental given the great affluence of motor skills (Castañer et al., 2018), which can be seen in all kinds of physical activities and sports. For example, in a 1x1 team sports situation, a player must decide quickly and instantly to overcome the opponent, knowing that an improper reading of the environment could mean losing possession of the ball (i.e., dysfunctional impulsiveness). On the contrary, a correct perception and decision, but sometimes risky, could lead to successful goal achievement (i.e., functional impulsiveness).

At the sporting level, the variables speed and efficiency play a fundamental role, especially in the information processing, decision-making and motor execution (Burnett et al., 2012). From this perspective, it seems logical to reflect what role impulsiveness plays in rapid, risky and effective decision-making, and consequently in motor performances.

The review of the scientific literature leads us to raise the question about whether impulsiveness in motor execution could be considered always “negative” or prejudicial. On the line of Dickman and Meyer (1988), we hypothesize that, on many occasions, the ability to act quickly and impulsively on risky situations that require effective motor responses are fundamental, not only in the field of Physical Activity and Sport but also in everyday life. Considering the interest in the patterns' detection of impulsiveness on adolescents and young adults' motor responses, we aim to study the relationship of impulsiveness, motor skills, motor laterality (Bishop et al., 2013; Castañer et al., 2018) and decision-making (Burnett et al., 2012) in staggered situations, through a design Mixed Methods (Castañer et al., 2013). Thus, the objective of this study is to deepen how impulsiveness affects athletes' motor responses by analyzing if impulsiveness generates proactive motor behaviors.

## Method

**Figure 1:**
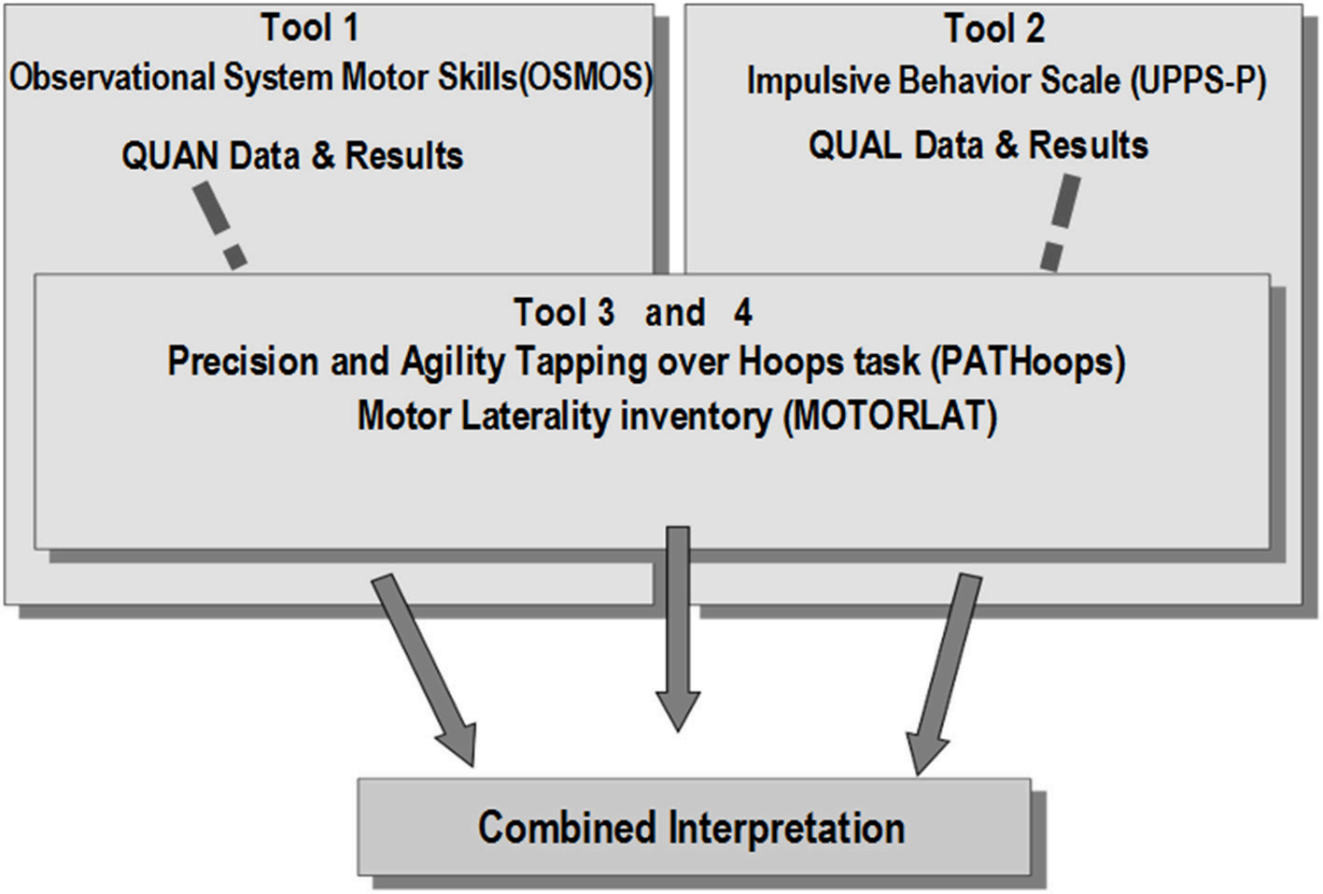
Embedded Mixed Methods design.

The Mixed Methods approaches are increasing especially in Physical Activity and Sport (Camerino et al., 2012; Castañer et al., 2013). For this reason, we conducted an embedded Mixed Methods design to merge the analysis of both impulsiveness and motor skills behaviors. As can be seen in Figure 1, the embedded design includes: (a) an adapted version of Observational System of Motor Skills (OSMOS) (Castañer et al., 2009, 2016) to observe the motor behaviors; (b) The Spanish version of Impulsive Behavior Scale (UPPS-*P*) to obtain the impulsiveness profiles; (c) The Precision and Agility Tapping over Hoops (PATHoops) (Castañer et al., 2018), to observe the decision-making in the temporo-spatial organization; and (d) Motor Laterality Inventory (MOTORLAT) (Castañer et al., 2010, 2018), to get the laterality profiles of motor skills performance.

### Participants

A total of 71 participants (53 males and 18 females) ranging in age from 18 to 24 years (*M*_age_ = 18.5 years; *SD* = 1.72) that regularly attended the Physical Education course were analyzed. They performed three staggered situations from closed to open motor tasks in classes. Previously, all participants were notified about the study, and they signed a written informed consent form to participate in the research. As for the observational methodology, the observation sessions were conducted in a natural context, specifically we observed the participants' development in the scholar sessions, therefore the institution has a consent form about image privacy that students enrolling at the school are required to sign. The study was approved by the ethics committee of Sports Medicine and Health of Catalan Counseling of Sport, Barcelona, Spain (code 10-2018-CEICGC).

### Materials

#### Impulsive Behavior Scale (UPPS-P)

The UPPS-P (Cyders et al., 2014) is used to measure impulsiveness facets. The tool, comprised of five facets and 20 items in total, evidenced good internal consistency across their subscales (Cyders et al., 2014). The five facets separately assess the following dimensions: (1) *Sensation Seeking*; (2) *Lack of premeditation*; (3) *Lack of perseverance*; (4) *Negative urgency;* and (5) *Positive urgency*. Each dimension comprises four items with a Likert scale from 1 to 4 (1: Strongly Agree, 2: Agree, 3; Disagree, and 4: Strongly Disagree). Three of these facets serve to identify ADHD disorder (Geurten et al., 2018). The Spanish version of UPPS-*P* (Cándido et al., 2012) was used in this study.

#### Observational System of Motor Skills (OSMOS)

An adaptation of the observation instrument OSMOS (Castañer et al., 2009, 2016) was used, with a minimal optimization of criteria (Table 1). The instrument includes eight criteria: (1) Appropriateness of motor responses (adequateness of the participant's response); (2) Stability (motor actions without displacement of the body, i.e., turns, jumps, and balances); (3) Locomotion (motor actions that require displacement of the body); (4) Manipulation (motor actions performed with material or other participants contact); (5) Space (changes of direction or height levels); (6) Zone (area where the participant moves); (7) Time (performing pauses and changes of rhythm); (8) Interaction (including dyadic, group or both interactive behaviors). Each criterion was expanded to build an exhaustive and mutually exclusive observation system tool that included, in total, 25 categories.

**Table 1:** An adaptation of the observation instrument OSMOS (Castañer et al., 2009, 2016).

**Criterion**	**Category**	**Code**	**Description**
Appropriateness of responses	Inappropriate motor responses	IMR	Motor actions and interactive behaviors that the participant performs unrelated to the task.
Stability	Support stability	SS	Motor skills that enable body balance to be maintained over one or several body support points, without producing locomotion (e.g., balancing actions).
	Elevation stability	ES	Motor skills that enable the body to get off the ground without locomotion (e.g., jumps).
	Axial stability	AS	Motor skills that enable body axes and planes to be varied from a fixed point, without producing locomotion (e.g., turns).
	Combination of Stability	COS	Combination of the previous criterion's categories.
Locomotion	Propulsion-stop locomotion	PSL	Motor skills that occur at the start and finish of a body movement through space.
	Sequential rebalance locomotion	SRL	Motor skills that enable displacement through the priority sequence of actions of the lower limbs segments (bipedal locomotion) or upper limbs (in inversion).
	Simultaneous coordinated locomotion	SCL	Motor skills that enable displacement through the combined action of all body segments (e.g., quadrupedal locomotion).
	Combination of Locomotion	COL	Combination of the previous criterion's categories.
Manipulation	Impact manipulation	IM	Motor skills in which certain body zones briefly contact with objects or other people.
	Conduction manipulation	CM	Motor skills in which certain segments handle (for a given period of time) objects or other people.
	Combination of Manipulation	COM	Combination of the previous criterion's categories.
Space	Change in spatial direction	CSD	Variations between the different levels of the horizontal component of displacement.
	Change of spatial level	CSL	Variations between the different levels of the vertical component of displacement (low or floor, middle or bipedal, upper or aerial work).
	Maintenance in the same space	MSS	The participant stands in the same area of the space.
	Combination of variations in body posture/gestures and spatial direction	CSP	Combination of the previous criterion's categories.
Zone	Central	CEN	The participant moves in the middle area of the space.
	Peripheral	PER	The participant moves in the external area of the space apart from the corners.
	Corner	COR	The participant moves in the vertices of the space.
Time	Change of Rhythm	CRY	When there is a clear observable tempo variation of a motor action.
	Pause	PAU	When the participant remains in a static position.
Interaction	Dyadic interaction	DYI	Synergy with a partner.
	Group interaction	GRI	Synergy with more than one other member that act together.
	Non-interaction	NIN	Inexistence of synergies.
	Combination of Interaction	COI	Combination of the previous criterion's categories.

#### Motor Laterality Inventory (MOTORLAT)

The MOTORLAT (Castañer et al., 2018) was used in the study to detect laterality profiles from motor skills performance. It consists of four criteria from OSMOS instrument (Castañer et al., 2009, 2012), but each criterion was expanded to build an exhaustive and mutually exclusive total of 30 items of fundamental and combined motor skills. The description of the four criteria are: (1) *locomotion skills*: actions that require the body to move from one point to another across space; (2) *stability skills*: actions that do not require the body to move from one point to another across space (i.e., jumping, balancing and turning); (3) *manipulation skills*: actions that require the manipulation of objects or other people with the limbs of the body; and (4) *combined skills*: actions that combine one or more of the locomotion, stability and manipulation skills criteria.

#### The Precision and Agility Tapping Over Hoops Task (PATHoops)

**Figure 2:**
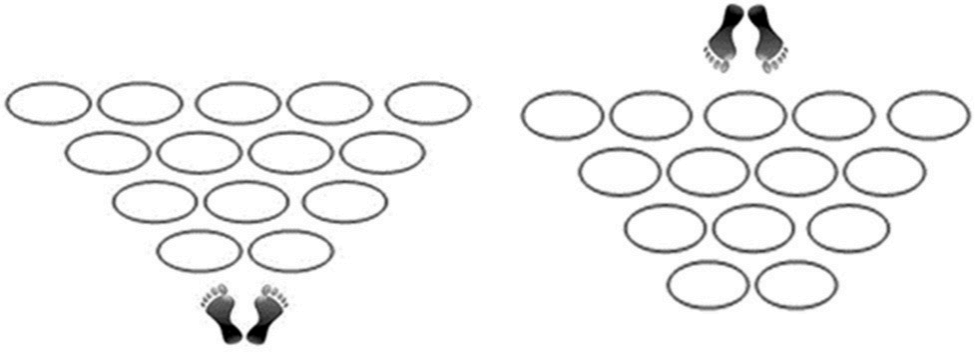
PATHoops Task. **(Left)** Starting position from the narrow side of PATHoops. **(Right)** Starting position from the wide side of PATHoops (Castañer et al., 2018).

The PATHoops (Castañer et al., 2018) consists of a task in which “participants, standing on both feet, were asked to perform a path by stepping in each of 14 hoops arranged in a triangular shape on the floor. In addition, participants were asked to perform the PATHoops task from both sides” (Castañer et al., 2018, p. 4). Figure 2 illustrates the position of the participants in front of the hoops' distribution. The task allows to observe the decision-making of participants during the entire task, that is, stepping quickly and exclusively into all the single 14 hoops. For instance, previous research (Castañer et al., 2018), applied to young athletes, has demonstrated that zigzag way, which is covering from one wing to the other wing of the triangular figure, is the optimal way to perform the task skillfully.

### Procedure

Firstly, we administered the short Spanish version of the UPPS-P (Cándido et al., 2012) in order to obtain the impulsiveness profiles of the participants. Likert values of the items 1, 4, 5, 7, 12, and 19 items from the short Spanish version of the UPPS-P scale were analyzed following the protocol of reverse data. Once the impulsiveness profiles were obtained, we proceed to register the staggered workouts that were introduced into three different normal Physical Education classes. The three workouts had a duration of 5 min each one and consisted of performing individual motor skills into the three following designs:

1. Different materials (e.g., balls, hoops, ropes) were placed together in different zones of the space. Participants could use these materials into the zones without interacting among them.
2. Same materials (e.g., balls, hoops, ropes) were in the same zone of the space and participants could use them and only group interaction was allowed.
3. Participants could use material and interaction in a freely way.

MOTORLAT inventory and PATHoops task were administered individually in following Physical Education classes to preserve the personal decision-making and exclude imitation on workouts' performance.

### Data Analysis

**Figure 3:**
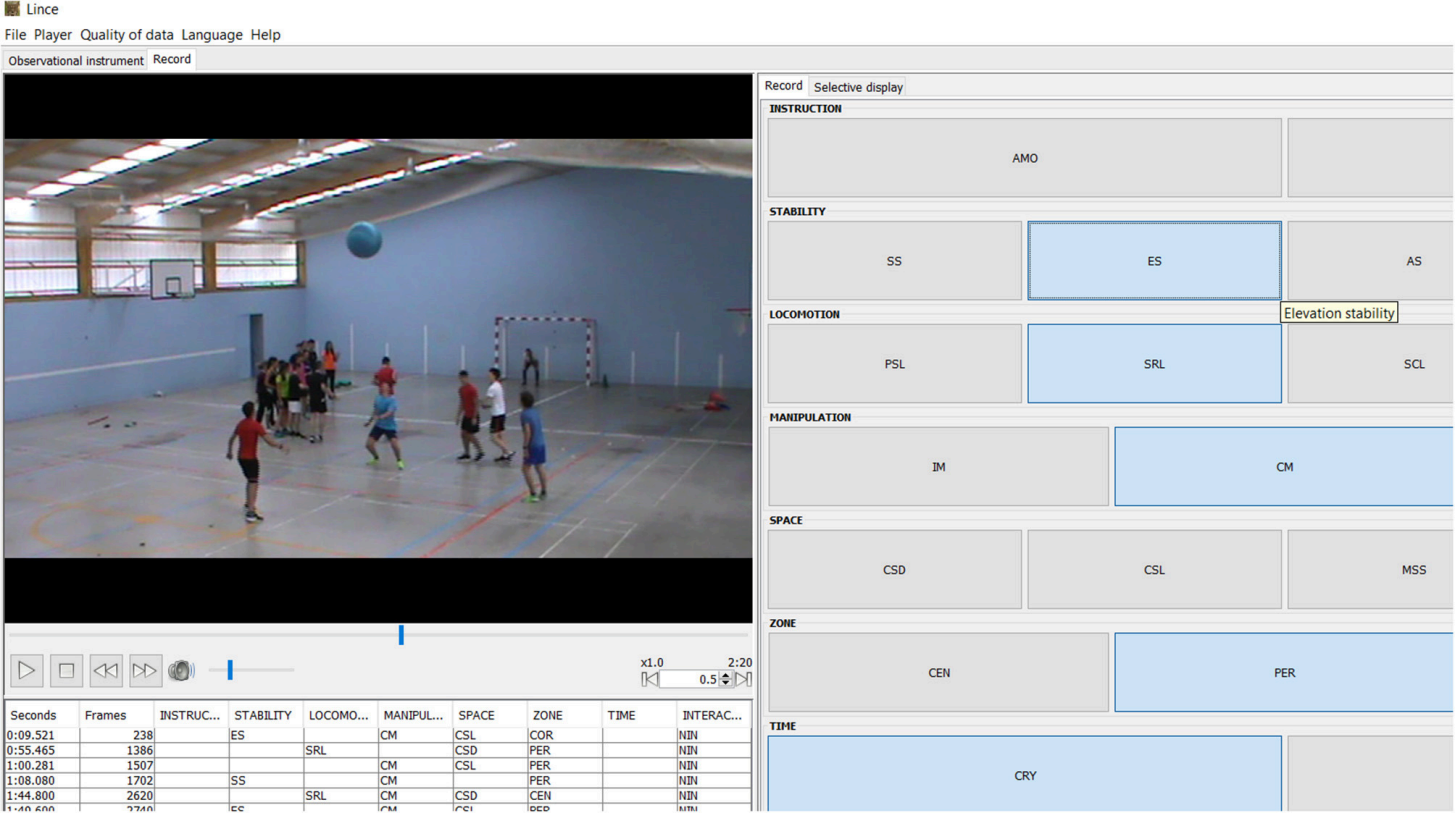
The screen of software LINCE (Gabin et al., 2012). Written informed parental consent was obtained for the publication of this image.

The OSMOS behavior coding tool and the videos of the staggered workouts were coded using LINCEv.1.2.1 (Gabin et al., 2012) by two experts on motor behavior. Intra-observer and inter-observer reliability were calculated in LINCE software before the full data set codification obtaining a kappa statistic of 0.95 and 0.91, respectively, which guarantees the interpretive rigor of the coding process. LINCE software is a highly useful software that facilitates the systematic observation of behaviors and integrates a wide range of functions such as coding, data quality check and conversion to several data extensions, which helps the exportation to several statistical analysis software. Figure 3 shows a screen capture of coding data in LINCE.

THEME software package (Magnusson et al., 2016) was used to T-pattern detection of observational data. THEME software is a data analysis research tool that has been increasing the interest of researchers for the last two decades to obtain T-patterns, which essentially uses an algorithm to compare all coded behaviors from the simplest to the most complex combinations. THEME software underlines the detection of a statistically significant chain of behaviors as has to deal with a great amount of behavioral events. To avoid that T-patterns are discovered only by chance, THEME works by randomizing and re-analyzing the original data repeatedly using the same search parameters (Casarrubea et al., 2015).

Laterality profiles were obtained by cluster analysis and subsequent correlational analyses were carried out as we have conducted in previous studies (Castañer et al., 2018). Internal assessment of this was done by correlational analysis between the motor skills of the MOTORLAT items (Table 3). As that previous study (Castañer et al., 2018), a contingency analysis was used to cross the limb dominance criteria from the MOTORLAT inventory and their relationships with the spatial orientation criteria from the PATHoops task.

## Results

### Impulsiveness Profiles

**Figure 4:**
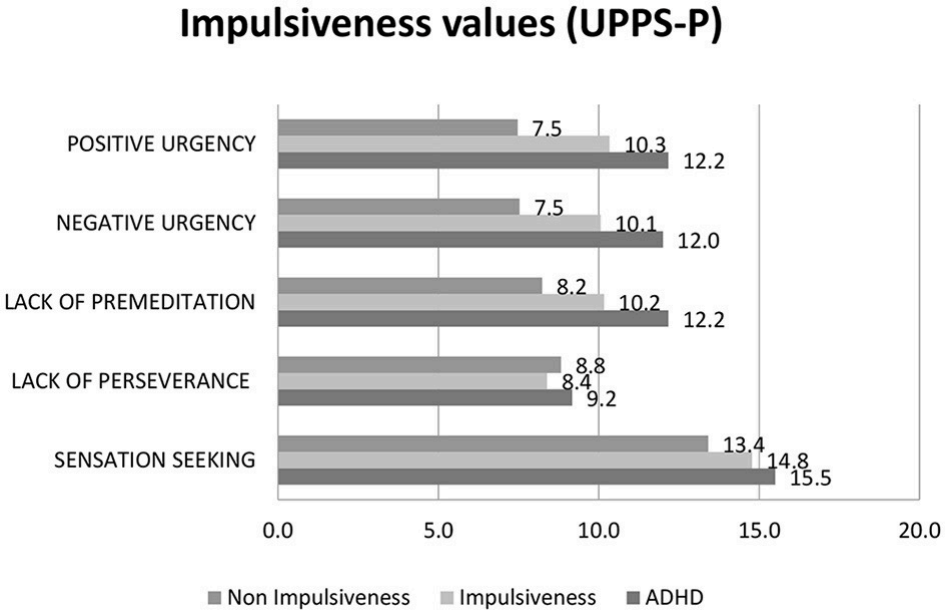
Values of the types of impulsiveness from the five distinct facets of the Impulsive Behavior Scale (SUPPS-*P*).

The values of SUPPS-*P*'s five facets revealed a total of 54 impulsiveness profiles of the total 71 participants. We differentiated three profiles: impulsiveness (*n* = 47); ADHD (*n* = 7); and non-impulsiveness (*n* = 17). Results showed a notable difference between impulsiveness and non-impulsiveness participants; mainly in the facets described by Geurten et al. (2018) namely lack of premeditation, positive urgency, and negative urgency (Figure 4).

The data obtained from item 9 of the UPPS-*P*, which refers to the joy of taking risks, and which belongs to the facet of sensation seeking, have shown that the majority of participants agreed with the enjoyment of taking risks. For instance, 71.4 and 57.4% of the participants with ADHD and with impulsiveness Strongly Agree, respectively. Moreover, impulsiveness (42.6%) and non-impulsiveness (58.9%) participants responded to Agree (Table 2).

**Table 2:** Item 9 UPPS- *P*, I quite enjoy taking risks vs. Impulsiveness type.

		**Impulsiveness type**			**Total**
		**Impulsiveness**	**ADHD**	**Non- impulsiveness**	
9.- I quite enjoy taking risks.	Disagree Strongly	0	0	0	0
	Disagree	0	0	4	4
	Disagree				
	Agree	20	2	10	32
	Strongly Agree	27	5	3	35
	Total	47	7	17	71

**Table 3:** Skilled foot vs. Impulsiveness types.

		**Impulsiveness types**			**Total**
		**Impulsiveness**	**ADHD**	**Non-impulsiveness**	
Foot used to kick the ball	Right	37	5	16	58
	Left	10	2	1	13
The foot that touches the ball	Right	33	5	15	53
	Left	14	2	2	18
Total		47	7	17	71

### Laterality Profile and Spatial Orientation

The motor skills of kicking and touching a ball with the feet, which were assessed by MOTORLAT inventory, showed differences between participants. Non-impulsiveness participants used mainly the right foot in both motor skills (94.1% for kicking a ball, and 88.2% for touching a ball). On the other hand, participants with impulsiveness and with ADHD showed greater use of the left foot. For instance impulsiveness participants reduced the use of right foot to 78.7% for kicking a ball and 70.2% for touching a ball, and ADHD participants decreased the use of right foot to 71.4% for both actions of kicking and touching a ball.

The administration of the PATHoops task revealed two interesting data, one related to the foot that the participants used to start the task (Table 4) and the description that they give to how to perform the entire task (Table 5). Table 4 shows that non-impulsiveness, impulsiveness and ADHD participants use the right foot 76.4, 53.1, and 71.4%, respectively.

**Table 4:** The foot that starts the Hoops task vs. Impulsiveness type.

		**Impulsiveness type**			**Total**
		**Impulsiveness**	**ADHD**	**Non-impulsiveness**	
Foot to start the task	Right	25	5	13	43
	Left	22	2	4	28
Total		47	7	17	71

**Table 5:** Decision making of the way in HOOPs vs. Impulsiveness types.

		**Impulsiveness types**			**Total**
		**Impulsiveness**	**ADHD**	**Non-impulsiveness**	
The Way of HOOPs	Zigzag way	32	2	10	54
	Other way	15	5	7	27
Total		47	7	17	71

Complementarily, Table 5 distinguishes the participants who complete the entire task by performing a zigzag way (non-impulsiveness by 58.8%; impulsiveness by 68%; and ADHD by 28.5%) from those who perform the task describing other ways (non-impulsiveness by 41.1%; impulsiveness by 31.9%; and ADHD by 71.4%).

### Motor Behavior T-Pattern Detection

**Figure 5:**
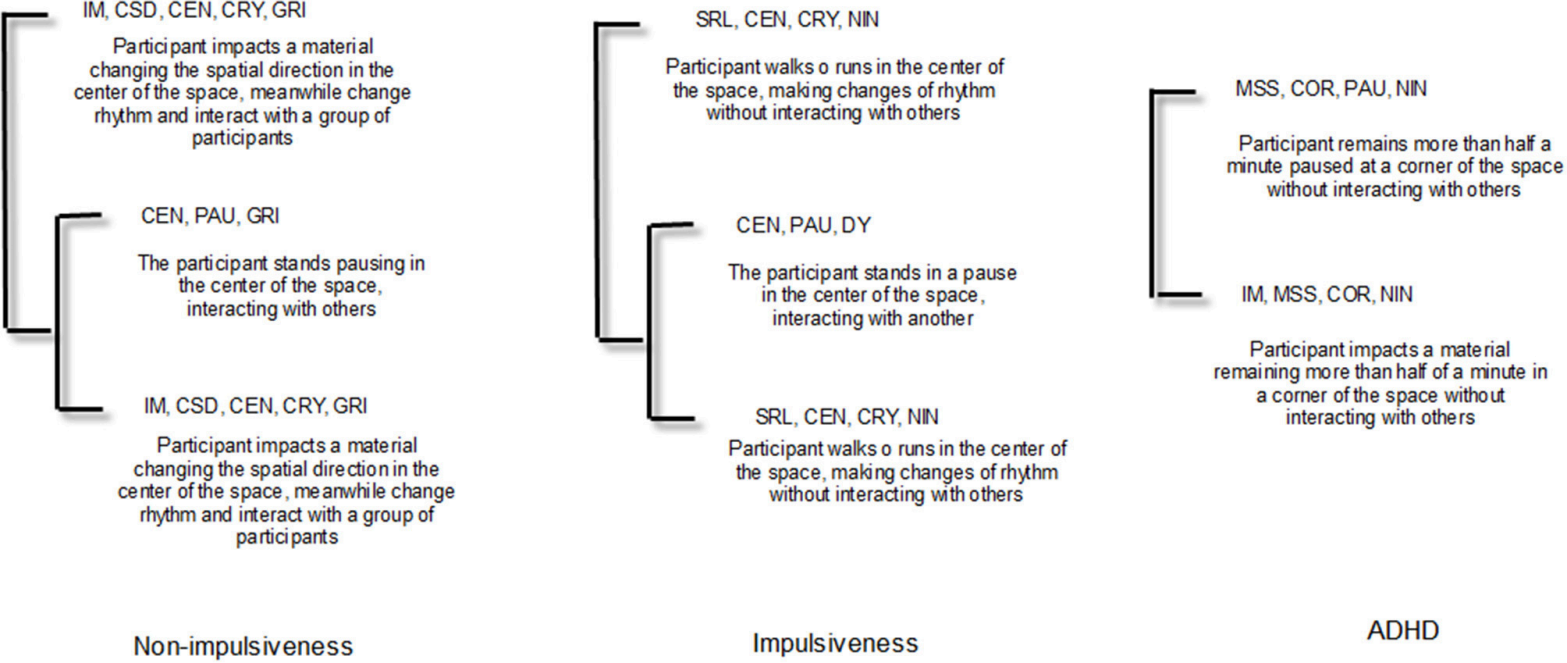
The three most relevant T-patterns of each type of impulsiveness.

From the total participants' data, we have only obtained T-patterns in the second workout. Thus, we selected the three most relevant—not due to its complexity but because of its clarity to the motor skills performed—to explain the motor behavior of each three levels of impulsiveness. We expose these three T-patterns regarding each type of impulsiveness (Figure 5).

## Discussion

The discussion section is structured following the order of the results of the different tools that have fulfilled the Mixed Methods approach (Castañer et al., 2013; Anguera et al., 2017), which was used to obtain a broader interpretation of impulsiveness profiles and workout performances of adolescents and young adults.

### Impulsiveness and Motor Behavior

The results from the Spanish version of UPPS-*P* scale showed a notable difference between the impulsiveness and the non-impulsiveness participants, mainly in the facets of lack of premeditation, positive urgency and negative urgency (Geurten et al., 2018). Most participants agreed with the idea to search for new experiences and sensation seeking. While participants with ADHD agreed with taking risks without taking care of the consequences, the other participants with impulsiveness and non-impulsiveness preferred to establish premeditation. These results corroborate with the studies of Cyders and Smith (2008) and Miller et al. (2003). The latter about young adults with ADHD that showed antisocial responses with sensation seeking without premeditation.

Questionnaires have certainly made a useful contribution in detecting the facets of impulsiveness. Nevertheless, to state how participants with diverse types of impulsiveness perform motor behaviors, they must be in a natural context (Anguera, 2003). In this sense, Anguera et al. (2012), Hudak et al. (2017) from the neurosciences field, argue that protocols developed on laboratories must be translated to real-world conditions. For this reason, this study was conducted on Physical Education settings, where an inventory of motor laterality, a task of decision-making, spatial orientation and three staggered motor situations were introduced.

#### Decision-Making in Body Laterality Uses and Spatial Orientation

Related to motor laterality profile of participants in the uses of the upper and lower limbs, MOTORLAT inventory revealed that only the motor skills of kicking and touching a ball with a foot showed differences between participants' impulsiveness types. Specifically, non-impulsiveness subjects used more times the right foot in both motor skills, and the impulsiveness and ADHD ones showed a higher percentage of preference for the left foot. These results support the scientific literature about left- and mixed- footedness associated with ADHD (Tran and Voracek, 2016). These authors found that mixed-footedness was not only probably associated with ADHD but also related to higher inattention and impulsiveness. Furthermore, mixed laterality can be a versatile and rich skill to perform successfully in multifaceted environments (Chapple and Johnson, 2007), and of interest to optimize the athletes' complex movements performance (Loffing et al., 2016). In this regard, previous research in football concluded that Lionel Messi—a left-footed top-player—is a good example of motor versatility as he uses successfully mixed laterality in several motor skills (Castañer et al., 2016, 2017a,b).

How can we know whether the mixed laterality optimizes or not motor behaviors? We obtained the answer to this question from the analysis of participants' decision-making used in PATHoops task, as data analysis show two interesting results: (a) the foot selected by participants to start the task and (b) the way described to perform the entire task throughout the 14 hoops. On the one hand, results obtained shows the similarity between the selected foot to start PATHoops task and MOTORLAT scores for kicking and touching a ball. For instance, non-impulsiveness participants used mainly the right foot, while the other participants chose sufficiently the left foot.

On the other hand, we consider that the participants' description of the way to perform the task, explains clearly the optimal decision-making on motor behavior to solve tasks, as in this task can be observed the decision-making of participants to complete the entire task that is, stepping quickly into all the 14 hoops without repeating anyone. As we stated, previous research demonstrated that young athletes use a zigzag way that is, covering from one wing to the other wing of the triangular figure, to perform the task skillfully (Castañer et al., 2018). In our research, results have shown that non-impulsiveness and impulsiveness participants decide sufficiently this zigzag way. On the contrary a high percentage of ADHA participants decided to perform another way, which is not so optimal to decide spatial orientation according to decision-making theories (Baker and Côté, 2003; Aguilar et al., 2018).

### Performing Motor and Interactive Patterns

Results from T-patterns were obtained mainly from the second workout (i.e., one T-pattern) but none from the first workout. The fact that the first workout did not provide any motor behavior T-pattern can be logically explained due to the rules of the situation: (a) the lack of interaction and (b) the constriction to use the material only in where it was placed. In these kinds of situations, participants did not know what to do and often spotted their motor actions in isolate ways without a sense. An aspect that corroborates with researches focused on motor interaction (Aguilar et al., 2018). Similarly, the third workout (based on full interaction and use of material) that appeared very creative or motor behavior enhancing, have not produced enough T-patterns. It seems that excess of materials, non-restrictions in the ways to use them and interacting with others are a detriment to the richness of motor behaviors (Castañer et al., 2011, 2016; Casarrubea et al., 2018).

Curiously, the T-patterns obtained from each one of impulsiveness types shows a diminishing use of interactive responses that go from (a) a continuous interaction in the group of non-impulsiveness participants; (b) a punctual dyadic interaction of impulsiveness participants to; (c) none interaction of ADHD participants. Additionally, T-patterns of non-impulsiveness and impulsiveness types offer richer combinations of motor behaviors than ADHD type. In respect to the temporal and spatial facets, non-impulsiveness and impulsiveness participants use the center of the space and perform changes of actions' rhythm. Contrarily, ADHD remained on a corner area and made pauses. All these characteristics could be related to the facet of negative urgency, which implies that impulsiveness responses are sometimes performed in an antisocial way (Cyders et al., 2007).

### Merging Motor and Cognitive Processes

Cognitive processes are related to motor mechanisms and both optimize our actions. In fact, studies using neuroimaging confirmed that people with brain injuries or development disorders have a fundamental interrelationship between the motor and cognitive progressions (see Diamond, 2000, for a review). For example, brain imaging studies have shown a strong functional coupling between brain regions, which traditionally it was thought to be sustained exclusively by cognitive processes or motor ones (Stoodley, 2012). Recently, Ithas beensuggested that cognition provides the basis for many different social-cognitive skills (Gallese et al., 2009).

Moreover, studies in cognitive neuroscience have been implying the existence of a common neural mechanism that could be responsible for the actions and understandings of intention skills in both humans and non-human primates. These findings have revealed that the cortical area related to the movement of the body, which was always confined to the role of simple action, programming, and execution plays a crucial role in complex cognitive skills such as understanding the intentions and objectives of the actions.

We consider that our study adds knowledge to those research by offering a Mixed Method approach that deepens how impulsiveness can enhance optimal motor performances. However, this study has the limitation of not disposing sufficient number of ADHD participants to corroborate with more type of responses.

## Conclusion

In this study, we pointed out a methodological and a substantive research aspect to fulfill practical implementations and theoretical approaches over impulsiveness, respectively.

Methodologically, professionals and researches should choose to apply more than a single tool (e.g., standard tests) as Mixed Methods research (Anguera et al., 2012, 2017; Camerino et al., 2012; Castañer et al., 2013) have ensured here a broader understanding of impulsiveness. In our specific case using the SUPPS-*P* scale, the MOTORLAT inventory, the PATHoops task, and the OSMOS behavior coding tool.

Related to the substantive aspect to fullfill theoretical approaches over impulsiveness, professionals such as psychologists, educators, and neuroscientists, should have in mind that impulsiveness, when not associated to deficits or disorders (e.g., ADHD), is far to be considerate negative and frequently optimize motor situations. In fact, sportsmen with impulsiveness characteristics enhance: (a) richness of motor skills and spatial interpretations (Castañer et al., 2018); (b) richness of motor patterns in open motor situations (Aguilar et al., 2018), as they enhance technical and tactical strategies in sport teams (Castañer et al., 2017a); (c) and interpersonal interaction (Kang et al., 2011). In sum, we would point out that cognitive processes are closely related to motor ones and both of them seem to improve the driving cognitive optimization that is acquired throughout evolutionary stages.

## Author Contributions

MC developed the project, supervised the design of the study, the method section and the drafting of the manuscript. QP was responsible for the review of the literature. JA and BE collected and codified the data. OC was responsible for the critical revision of the content and data analysis. TF supervised the drafting of the manuscript. All authors approved the final, submitted version of the manuscript.

### Conflict of Interest Statement

The authors declare that the research was conducted in the absence of any commercial or financial relationships that could be construed as a potential conflict of interest. The handling editor declared a past co-authorship with one of the authors MC.
